# Aerobic Exercise and Endocannabinoids: A Narrative Review of Stress Regulation and Brain Reward Systems

**DOI:** 10.7759/cureus.55468

**Published:** 2024-03-04

**Authors:** Subir Gupta, Ambadasu Bharatha, Damian Cohall, Sayeeda Rahman, Mainul Haque, Md Anwarul Azim Majumder

**Affiliations:** 1 Physiology, Faculty of Medical Sciences, The University of the West Indies, Cave Hill Campus, Bridgetown, BRB; 2 Pharmacology, Faculty of Medical Sciences, The University of the West Indies, Cave Hill Campus, Bridgetown, BRB; 3 Pharmacology, School of Medicine, American University of Integrative Sciences, Bridgetown, BRB; 4 Pharmacology and Therapeutics, Karnavati Scientific Research Center (KSRC) School of Dentistry, Karnavati University, Gandhinagar, IND; 5 Pharmacology and Therapeutics, National Defence University of Malaysia, Kuala Lumpur, MYS; 6 Medical Education, Faculty of Medical Sciences, The University of the West Indies, Cave Hill Campus, Bridgetown, BRB

**Keywords:** acute exercise, aerobic training, stress management, endocannabinoids, mood, hpa axis, cannabinoid receptors, endocannabinoid system, 2-acylglycerol, n-arachidonoyl-ethanolamine

## Abstract

Aerobic exercise is a widely adopted practice, not solely for enhancing fitness and reducing the risk of various diseases but also for its ability to uplift mood and aid in addressing depression and anxiety disorders. Within the scope of this narrative review, we seek to consolidate current insights into the endocannabinoid-mediated regulation of stress and the brain's reward mechanism resulting from engaging in aerobic exercise.

A comprehensive search was conducted across Medline, SPORTDiscus, Pubmed, and Scopus, encompassing data available until November 30, 2023. This review indicates that a bout of aerobic exercise, particularly of moderate intensity, markedly augments circulating levels of endocannabinoids - N-arachidonoyl-ethanolamine (AEA) and 2-acylglycerol (2-AG), that significantly contributes to mood elevation and reducing stress in healthy individuals.

The current understanding of how aerobic exercise impacts mental health and mood improvement is still unclear. Moderate and high-intensity aerobic exercise modulates stress through a negative feedback mechanism targeting both the hypothalamus-pituitary-adrenal (HPA) axis and the sympathetic nervous system, thereby facilitating stress regulation crucial role in endocannabinoid synthesis, ultimately culminating in the orchestration of negative feedback across multiple tiers of the HPA axis, coupled with its influence over cortical and subcortical brain structures. The endocannabinoid has been observed to govern the release of neurotransmitters from diverse neuronal populations, implying a universal mechanism that fine-tunes neuronal activity and consequently modulates both emotional and stress-related responses. Endocannabinoids further assume a pivotal function within brain reward mechanisms, primarily mediated by CB_1_ receptors distributed across diverse cerebral centers. Notably, these endocannabinoids partake in natural reward processes, as exemplified in aerobic exercise, by synergizing with the dopaminergic reward system. The genesis of this reward pathway can be traced to the ventral tegmental area, with dopamine neurons predominantly projecting to the nucleus accumbens, thereby inciting dopamine release in response to rewarding stimuli.

## Introduction and background

Over numerous years, studies have focused on investigating the impact of exercise on human cognition and health and delving into the underlying mechanisms through which physical activity influences well-being [1, 2]. In contemporary times, aerobic exercise is widely endorsed to enhance cognitive functions and elevate mood. While the physiological foundations of cardiorespiratory adaptations are well-established, the mechanisms that underlie the amelioration of depression, anxiety, and mood enhancement through physical activity remain inadequately elucidated [3]. The contribution of resistance exercise to positive mood alterations is a subject of inquiry, with specific studies suggesting that resistance exercises, such as weightlifting, may rival aerobic exercise in their mood-elevating efficacy. However, the mechanism of mood elevation can vary between aerobic and resistance exercises [2,4,5]. Furthermore, alongside aerobic and anaerobic regimens, mind-body practices like yoga [6] and specialized exercises such as whole-body vibration [7, 8] hold potential for enhancing mental well-being.

Since their identification in the 1990s, endogenous cannabinoids [endocannabinoids (eCBs), have captivated the interest of researchers in the fields of health and medicine, particularly regarding their potential impact on human well-being and health. The pioneering experimental study conducted by Sparling and colleagues in 2003 marked a significant milestone by revealing the influential role of eCBs in enhancing mood, providing pain relief, and contributing to the cardiorespiratory and metabolic advantages associated with physical exercise [9]. There is an intricate relationship between stress and eCBs, which involves activating the endocannabinoid system (ECS) as a reaction to physiological and emotional stress factors [10]. Endocannabinoids, the lipid-derived signaling molecules synthesized from neuronal membranes, are implicated in the mood-enhancing effects of acute aerobic exercise. The eCBs interact with cannabinoid receptors (CBRs) in various brain regions, orchestrating functions that are not fully understood. Diverse forms of aerobic exercise elicit an elevation in circulating levels of two prominent eCBs - N-arachidonoyl-ethanolamine (AEA, also known as anandamide) and 2-acylglycerol (2-AG) [11]. These eCBs bind to cannabinoid receptors (CB_1_and CB_2_) in the central nervous system and peripheral tissues. This binding regulates processes such as mood, appetite, and immune response. As retrograde messengers, eCBs influence neurotransmitter release, aiding in adapting the nervous system to stress and preserving homeostasis [12]. Increased AEA circulating levels during intense acute exercise may play a role in the immediate sense of well-being, providing insights into the phenomenon of exercise addiction that arises from repeated participation in such activities [13].

Certain scholars posit that the mood elevation induced by aerobic exercise might be an evolutionary adaptation that supported the survival of early humans amidst a challenging environment. This is attributed to the pain-numbing properties of eCBs released during physical exertion, enabling our ancestors to cover substantial distances during hunting and foraging [14]. Recent advancements in experimental evolution, exemplified by a selection of experiments conducted with rodents, propose that the rewards triggered by eCBs in response to exercise could have been a subject of natural selection. This may elucidate the propensity for habitual engagement in voluntary exercise observed in humans and across various mammalian species [15]. The escalation of circulating AEA levels during intense acute exercise potentially contributes to an immediate sense of well-being, shedding light on the phenomenon of exercise addiction resulting from repetitive engagement in such activities [16].

A burgeoning body of evidence in recent times underscores the involvement of the ECS in the central regulation of stress responses. However, the precise role of eCBs signaling within the phases of hypothalamus-pituitary-adrenal (HPA) axis regulation and the specific neural loci governing this regulation remain less than definitively delineated [17]. Various cerebral regions, including the hippocampus, amygdala, and hypothalamus, are thought to participate in eCBs-mediated stress modulation through the HPA axis [17].

This present narrative review endeavors to delve into the interplay between aerobic exercise and eCBs, in addition to exploring potential advantages associated with bouts of aerobic exercise and endurance training concerning stress management and the brain's reward system. Furthermore, it probes the ECS and its signaling mechanisms, elucidates the ramifications of aerobic exercise on HPA axis activity for physiological stress regulation, and provides an updated perspective on the conceivable neural circuits underpinning reward and mood elevation as an outcome of engaging in aerobic exercise.

## Review

Methods and materials

We conducted literature searches utilizing databases, e.g., Medline, SPORTDiscus, Pubmed, and Scopus, encompassing data available until June 30, 2023. The keywords used were: "Aerobic Exercise," "Endocannabinoids," "Stress Regulation," "Brain Reward Systems," "Endocannabinoid system," and "Cannabinoid receptors." Our review encompassed original studies, short communications, commentaries, editorials, perspectives, and reviews. Furthermore, we meticulously searched through the reference lists of relevant articles to identify additional sources. Additionally, we manually searched for relevant articles and consulted the websites of various professional associations and national/international scientific organizations. We established inclusion criteria encompassing studies focusing on human subjects, published in peer-reviewed journals, and written in English. Exclusion criteria comprised studies involving animal models, non-English language publications, and those lacking rigorous methodology.

Endocannabinoids and endocannabinoid-like compounds

The eCBs are lipid-derived compounds based on arachidonic acid, synthesized within neuronal membranes [18,19]. Arachidonic acid represents our body's most active ω-6 polyunsaturated fatty acid (PUFA). Two varieties of eCBs have been recognized as neurotransmitters: 2-AG and AEA. AEA falls into the N-acyl ethanolamines (NAEs) category, whereas 2-AG is classified as a monoacylglycerol [20-22]. The NAE family also encompasses N-oleoylethanolamine (OEA), an appetite-suppressing compound, and N-palmitoylethanolamine (PEA), which possesses anti-inflammatory and anti-proliferative properties [23]. Both OEA and PEA are categorized as eCB-like compounds rather than genuine eCBs, as they generally do not bind to cannabinoid receptors (CBRs) yet exhibit some cannabimimetic effects through the activation of alternative molecular targets [23].

Endocannabinoids are not stored within vesicles; they are promptly synthesized in response to Ca^2+^ influx upon neuronal depolarization. The concentration of 2-AG in the brain is approximately 200 times higher than that of AEA [24]. Endocannabinoids function as retrograde synaptic messengers, traversing a synapse after release and binding to presynaptic CBRs to hinder further neurotransmitter release. Several hypotheses have been suggested to explain how AEA is transported across the plasma membrane. These include the possibility of passive diffusion, a specific carrier that enables bidirectional transport known as an endocannabinoid transporter (eCBT), and the process of caveolae-associated endocytosis [25,26].

Cannabinoid receptors

The primary cannabinoid receptors, CB_1_ and CB_2_, have been discovered. These receptors are predominantly situated on presynaptic nerve terminals. CB_1_ receptor (CB_1_R) exhibits extensive expression across various brain regions [27, 28] and stands as the most abundant G-protein coupled receptor (GPCR) within the brain [29, 30]. CB_2_ receptor (CB_2_R), on the other hand, is mainly present in immune tissues' inflammatory cells and exhibits lower densities in the brain [29]. CB_1_R and CB_2_R have been cloned [31,32], cementing their roles as primary targets for eCBs [29,30].

Although the affinity of 2-AG for CB_1_R is lower than that of AEA, it can activate CB_2_R alongside CB_1_R. Despite having a lower affinity for CB_1_R, it is noteworthy that 2-AG acts as a full agonist, in contrast to AEA, which is a partial agonist [33]. Notably, while CB_2_R maintains its conformation upon activation, CB_1_R undergoes more extensive conformational changes in response to agonists. This heightened flexibility in CB_1_R during modulation enhances its capacity to respond to various ligands compared to CB_2_R [34].

Beyond CB_1_R and CB_2_R, eCBs may also interact with other receptors and ion channels, including at least three orphan GPCRs such as GPR55, GPR18, GPR119, as well as peroxisome proliferator-activated receptors (PPARs), transient receptor potential (TRP) channels like the transient receptor potential vanilloid 1 (TRPV1) cation channel, GABA receptors, and calcium, potassium, and sodium channels [35,36]. CB_1_R and CB_2_R are linked to G_i/o_ proteins, thereby inhibiting adenylyl cyclase and activating different members of the mitogen-activated protein kinase (MAPK) family [37,38]. In humans, two variants of the CB_2_R receptor have been identified. One variant is mainly found in the testes and, to a lesser extent, in the brain's reward areas, while the other is primarily found in the spleen and brain at reduced levels [39].

AEA, but not 2-AG, notably binds to TRPV1 (or Ca^2+^) channels. The PPARs constitute a family of nuclear receptors, encompassing PPAR_α_, PPAR_γ_, and PPAR_β/δ_, acting as transcription factors that regulate the expression of numerous target genes involved in metabolism, immune response, cell differentiation, and various other cellular changes and adaptive responses.

Endocannabinoid system and expanded endocannabinoid system or endocannabinoidome

**Figure 1:**
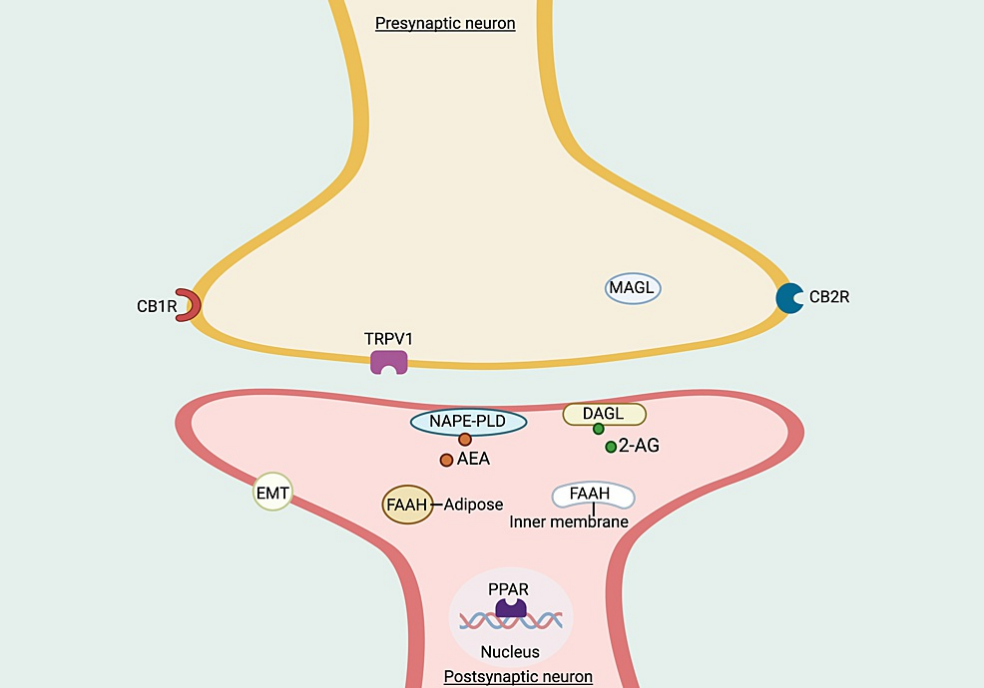
Illustration depicting the components of the endocannabinoid system Notes: AEA: N-arachidonoyl-ethanolamine; 2-AG: 2-arachidonoylglycerol (2-AG); CB_1_R: cannabinoid receptor type 1; CB_2_R: cannabinoid receptor type 2; DAGL: diacylglycerol lipase; EMT: endocannabinoid membrane transporter; FAAH: fatty acid amide hydrolase; MAGL: monoacylglycerol lipase; NAPE-PLD: N-acylphosphatidylethanolamine-phospholipase D; PPAR: nuclear peroxisome proliferator-activated receptor; TRPV1: transient receptor potential vanilloid type 1. This figure has been drawn with the premium version of BioRender (https://www.biorender.com/ Accessed date February 6^th^, 2024) with License Agreement No.: CW26FIBYOG. Image Credit: Subir Gupta

The ECS comprises eCB, CBRs, and enzymes responsible for synthesizing and breaking eCB compounds (Figure 1). The ECS, fully elucidated in the 1990s, encompasses two ligands - AEA and 2-AG, two CBRs, CB_1_R and CB_2_R, and five enzymes involved in synthesizing and degrading these two ligands. The primary enzymes responsible for the biosynthesis of these two ligands include N-acyl phosphatidylethanolamine phospholipase D (NAPE-PD) for AEA synthesis, diacylglycerol lipase-alpha, and diacylglycerol lipase-beta (DAGLs) for 2-AG synthesis, as well as fatty acid amide hydrolase (FAAH) for AEA breakdown, and monoacylglycerol lipase (MAGL) for 2-AG degradation [40].

Nowadays, the endocannabinoidome or expanded ECS encompasses 23 components. This extended framework includes 7 membrane-bound receptors and 3 enzymes, along with 4 cytoplasmic enzymes and 3 transporters (FAAH being associated with the endoplasmic reticulum), as well as a nuclear transcription factor and PPAR, which directly engage with eCBs [41]. Despite this comprehensive concept, the term "endocannabinoidome" remains relatively uncommon among clinicians and researchers and is often called the expanded ECS.

Synthesis and degradation of eCBs

Synthesis

**Figure 2:**
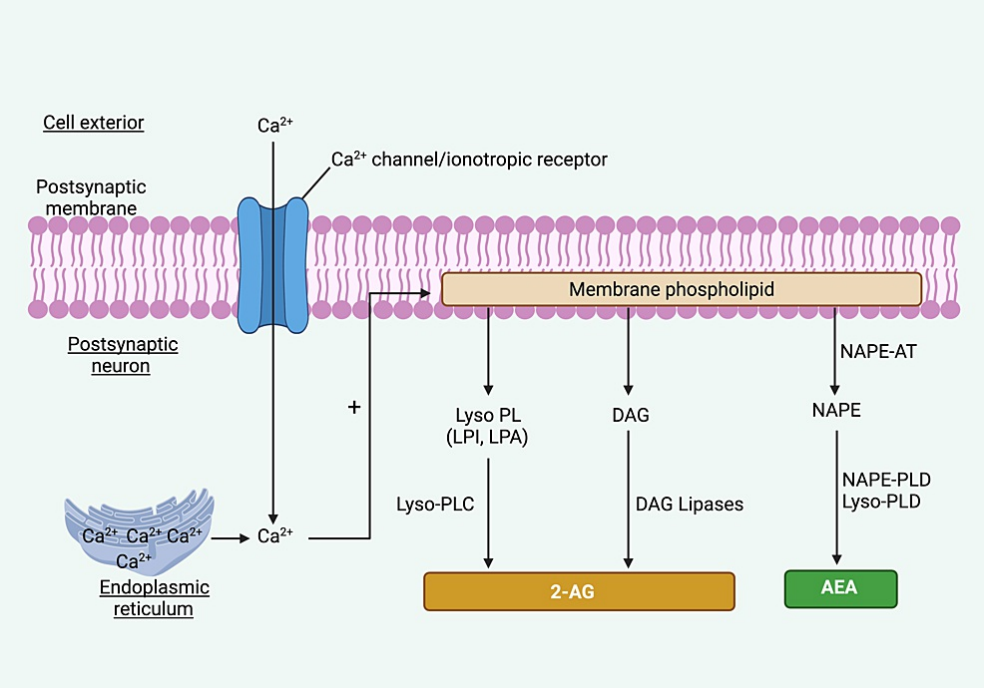
Diagrammatic depiction elucidating the biosynthesis of endocannabinoids Notes: 2-AG: 2-Arachidonoylglycerol, AEA: Arachidonoylethanolamine, DAG: Diacylglycerol, LPA: Lysophosphatidic acid, LPI: Lysophosphatidylinositol, NAPE: N-Acylphosphatidylethanolamine, NAPE-PLD: N-Acylphosphatidylethanolamine-specific phospholipase D, NAPE-AT: N-acylphosphatidylethanolamine acyltransferase, Lyso-PLC: lysophospholipase C, Lyso PL: lysophospholipid, Lyso-PLD: lysophospholipase D, LPI: lysophosphatidylinositol. This figure has been drawn with the premium version of BioRender (https://www.biorender.com/ Accessed date January 25^th^, 2024) with License Agreement No.: QW26DRZ3IS. Image Credit: Subir Gupta

Endocannabinoid precursors within lipid membranes undergo enzymatic transformation to generate eCBs. AEA and 2-AG are produced as a response to neuronal depolarization and/or the influx of Ca^2+^, accomplished through the cleavage of membrane phospholipids. eCBs form on a 'need-to-use' basis by post-synaptic neurons (Figure 2).

The metabolic pathways of 2-AG and AEA are fundamentally distinct. The synthesis of 2-AG is well-documented, whereas the mechanisms behind AEA synthesis remain less comprehended. 2-AG synthesis transpires via three principal routes from membrane phospholipids containing arachidonic acid - (i) through diacylglycerol (DAG) lipase, (ii) from 2-acyl lysophosphatidic acid (LPA) via 2-LPA phosphatase, and (iii) originating from 2-acyl lysophosphatidylinositol (LPI) via lysophospholipase C [37,42]. AEA is synthesized from arachidonic acid through a two-step process facilitated by N-acylphosphatidylethanolamines (NAPE)-specific phospholipase D (NAPE-PLD). Three distinct and independent mechanisms have been identified as contributors to AEA generation [37,42]; however, the primary pathway accountable for neuronal AEA synthesis remains undisclosed.

Degradation

**Figure 3:**
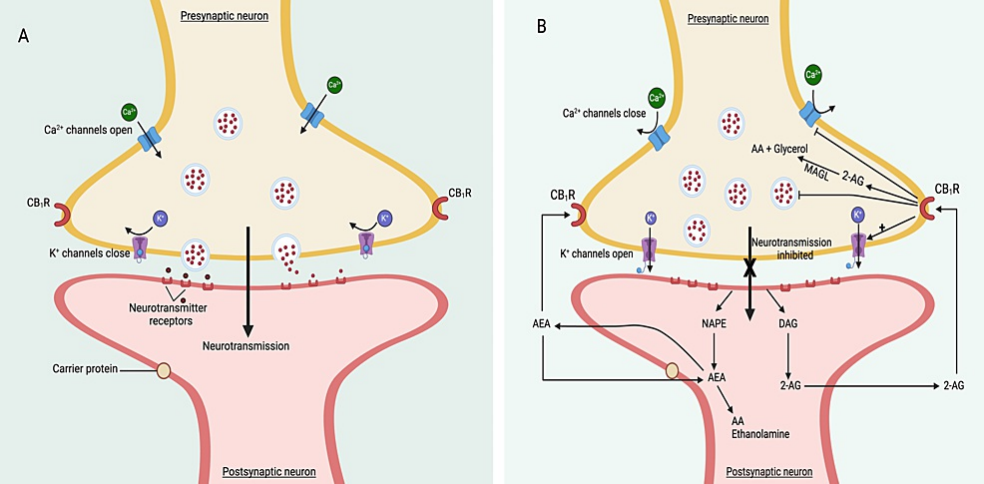
Diagrammatic depiction of the eCBs signaling system. Notes: In conditions devoid of exercise or stress, the uninterrupted flow of synaptic transmission persists, primarily attributed to the paucity or nearly negligible presence of endocannabinoids (eCBs) (A). However, a substantial surge in eCB secretion emanates from the post-synaptic membrane upon engaging in aerobic exercise of requisite intensity. These eCBs undergo retrograde diffusion, subsequently binding to CB_1_Rs situated upon the presynaptic membrane. This interaction culminates in the suppression or blockade of synaptic transmission (B). AEA: Anandamide, 2-AG: 2-Arachidonoylglycerol, CB1Rs: Cannabinoid Receptor Type 1, FAAH: Fatty Acid Amide Hydrolase, MAGL: Monoacylglycerol Lipase, COX-2: Cyclooxygenase-2, NAPE: N-acyl phosphatidylethanolamine phospholipase, DAG: Diacylglycerol, AA: Arachidonic acid; MAGL: Monoacylglycerol Lipase. This figure has been drawn with the premium version of BioRender (https://www.biorender.com/ Accessed date January 25^th^, 2024)with License Agreement No.: CT26DRZB1X (A) and XI26DRZCNW (B). Image Credit: Subir Gupta

The functional duration of eCBs within the synaptic space is contingent upon their processing via distinct enzymatic pathways. A pivotal catabolic player for AEA is fatty acid amide hydrolase (FAAH), which enzymatically cleaves AEA into ethanolamine and arachidonic acid [43,44]. The degradation of 2-AG primarily occurs under the influence of monoacylglycerol lipase (MAGL), yielding glycerol and arachidonic acid as byproducts [44,45]. Moreover, the arachidonoyl component of 2-AG can undergo oxygenation via cyclooxygenase (COX)-2 and lipoxygenases [46] (Figure 3).

Signaling of the endocannabinoid system

The modus operandi by which eCBs interact with their receptors (Figure 3) unveils a dynamic process: eCBs synthesized within post-synaptic neurons exert retrograde action pre-synaptically at CB_1_R [47]. Evidence suggests eCBs can also engage localized CB_1_R post-synaptically [48,49]. In essence, the activation of CB_1_R by eCBs diminishes the likelihood of neurotransmitter release through a multifaceted interplay, encompassing the inhibition of Ca^2+^ influx and the activation of K^+^ channels [47-50]. The effects culminate as eCBs undergo reuptake followed by degradation: 2-AG, facilitated by pre-synaptic MAGL, and AEA, orchestrated by post-synaptic FAAH, as earlier mentioned. Furthermore, a notable proportion (45-48%) of synapses across diverse brain regions adhere to a tripartite architecture, involving a pre-synaptic neuron, a post-synaptic neuron, and often an astrocyte-a glial cell that bears CB_1_R expression [48].

It has been proposed that eCBs are cleared from the synaptic realm via an uptake mechanism followed by enzymatic hydrolysis [26], akin to the process observed with other neurotransmitters or neuromodulators, such as the well-documented instance of dopamine reuptake by the dopamine transporter (DAT). Despite the eCBs membrane transporters not being fully characterized to date, their elucidation is in progress, as indicated by studies [25,51].

Acute aerobic exercise and the endocannabinoid system

Aerobic exercise engenders notable positive psychological changes, including mood elevation, stress reduction, and anxiolytic and antidepressant effects. Generally, a session of aerobic exercise has consistently shown associations with reductions in state anxiety and physiological arousal that endure for 2 to 4 hours [50]. The first experimental study aiming to unravel the influence of physical activity on eCBs in humans was conducted in 2003 by Sparling and colleagues [9]. Their analysis revealed increased plasma AEA content following 45 minutes of moderate-intensity exercise on a treadmill or cycle ergometer. Subsequent human investigations have corroborated heightened blood concentrations of AEA, though intriguingly not of 2-AG, after aerobic exercise durations of 30 to 45 minutes and up to 5 hours [15,52-54].

This mood-modifying effect of endurance exercise has been postulated to have conferred survival advantages to humans in hostile environments and during prolonged food-search activities, all without succumbing to excessive fatigue [13]. The resulting euphoric state, often dubbed the "runner's high," accounts for humans' and other animals' inherent inclination towards voluntary exercise. Notably, exercise-induced eCB activity in humans seems to be subject to modulation by exercise intensity [15,52,53], aligning with the understanding that the neurobiological repercussions of exercise are intricately linked with its intensity.

The optimal intensity of aerobic exercise that elicits a maximal eCB response remains a topic of ongoing debate. Brellenthin et al. [55] documented the most pronounced elevation in levels of AEA and 2-AG within the cohort engaged in high-intensity exercise. Conversely, Sparling et al. [9] observed significant increases in plasma AEA concentrations during workouts of moderate intensity, corresponding to 70% to 80% of the maximal heart rate (HR_max_). Raichlen and colleagues [15] demonstrated that, in both humans and dogs, AEA levels exhibit enhancement during moderate exercise intensities (70% of maximum heart rate in humans) rather than at lower intensities (45% of HR_max_). Alterations in eCBs and their analogs were observed to have a positive correlation with the intensity of the exercise (measured as a percentage of VO_2peak_) and adiposity of the individuals [54].

Although the precise relationship between aerobic exercise intensity and eCB levels remains not fully elucidated, insights emerge from a study by Raichlen et al. [56]. This study revealed post-exercise increments in AEA blood levels following slow jogging and medium-intensity workouts among self-reported fit, healthy adult men and women, in contrast to pre-exercise values. In essence, aerobic exercise elicits activation of the ECS within a specific range of exercise intensities. The research underscores the significance of considering exercise intensity when investigating the neurobiological effects of eCB signaling, given that exceedingly high or exceedingly low exercise intensities might not evoke eCB activity [54,56].

Aerobic training and the response of the endocannabinoid system

The conclusions drawn from investigations into the impact of aerobic training on mood elevation are marked by a degree of ambiguity. A growing curiosity centers on comprehending the ramifications of aerobic exercise on an individual's psychological state, particularly in terms of mood, anxiety, and depression, both at rest and after submaximal and maximal exertion. Nevertheless, a prevailing consensus asserts that the psychological shifts induced by chronic exercise are less profound than the effects manifested through acute aerobic activity [3,57]. Even in cases where alterations in psychological traits are absent, momentary enhancements in psychological states can ensue from acute exercise.

The research underscores that individuals experiencing moderate depression may observe significant psychological ameliorations after engaging in regular exercise. Conversely, shifts within standard samples tend to be of a lesser magnitude or may not manifest at all [58,59]. In essence, exercise tends not to substantially enhance psychological well-being among individuals initially within the typical spectrum of depression and anxiety.

In humans, consistent observations reveal that acute aerobic exercise yields a surge in circulating AEA [53,60]. Notably, Gasperi et al. [61] unveiled augmented up-regulation and activity of FAAH, a pivotal enzyme governing AEA degradation, within lymphocytes of physically active young men compared to their sedentary counterparts. This points toward heightened peripheral AEA metabolism concurrent with elevated exercise levels. An intriguing facet lies in the impact of AEA on mitochondrial function and oxidative metabolism pathways [62,63]. Such insights lead one to speculate about potential correlations between alterations in AEA levels and changes in cardiorespiratory fitness. However, studies investigating the exact associations between exercise training, cardiorespiratory fitness, and AEA remain relatively limited. Furthermore, a study examining male runners displaying symptoms of exercise addiction divulged lower plasma AEA concentrations both at rest and post-acute exercise when juxtaposed with non-addicted peers [64]. Similarly, Jurado-Fasoli et al. [54] demonstrated that engaging in moderate to vigorous aerobic and resistance exercises for 24 weeks led to a decrease in the blood levels of eCBs among young adults who previously led a sedentary lifestyle.

Stress regulation through exercise-mediated endocannabinoid modulation

Stress is characterized as a state in which the equilibrium of homeostasis is jeopardized by external or internal stimuli of potential harm [65]. In the absence of stress, normal AEA levels are maintained, stabilizing glutamate activity on basolateral amygdala (BLA) neurons without impacting the HPA axis [17]. The stress response comprises two facets: (i) the release of noradrenaline and adrenaline by the sympathetic nervous system (SNS) and (ii) the secretion of cortisol through the HPA axis. While CB_1_R signaling is recognized for its capacity to hinder noradrenaline release by the SNS, a more profound understanding prevails concerning its regulation of the HPA axis. The HPA axis and SNS function in tandem, simultaneously enhancing adaptive responses to immediate stressors and mitigating future threats [65-67]. If restoration of homeostasis is impeded upon cessation of the stressor or prolonged disruption occurs, these responses, such as heightened cortisol levels or persistent sympathetic activation, can assume a pathogenic character and contribute to the development of diseases.

Clinically, the stress response can be assessed by evaluating either or both of its components. In response to acute stress, the HPA axis is activated to safeguard the organism's survival [17,52]. Cortisol levels are typically examined to gauge HPA axis activity, with acute stress correlating with elevated levels. During stress, activated FAAH breaks down AEA in all brain regions. Simultaneously, 2-AG levels in the amygdala and hypothalamus either remain unchanged or decrease. This results in compromised AEA/CB_1_R signaling, leading to increased glutamate input onto basolateral amygdala (BLA) neurons [17]. The eCBs signaling system is a pivotal modulator of the stress response, akin to its role in aerobic exercise. It is crucial in facilitating a seamless return to the non-stressed state. The ECS restrains the extent of the stress-induced response associated with exercise, aids in normalizing the HPA axis to baseline levels, and contributes to the habituation of the exercise-induced response through aerobic training [68,69].

Moreover, it directly counteracts stress-associated processes encompassing fear, anxiety, depressive tendencies, inflammation, and heightened sensitivity to pain. Simultaneously, the ECS promotes behaviors curbed by the stress response, such as feeding and sleep patterns. In chronic stress, the inadequate elevation of 2-AG/CB_1_R signaling caused by various stressful situations leads to the downregulation of CB_1_R signaling in the medial prefrontal cortex (PFC) and the hypothalamus, creating a state known as a "hypocannabinoid state" [52].

The Involvement of Endocannabinoids in Modulating Sympathetic Nervous System Stimulation

Empirical support for eCBs-driven presynaptic inhibition within the sympathetic nervous system is notably limited [70]. Nonetheless, a select few studies have indicated that the activation of peripheral presynaptic CB_1_R leads to the inhibition of noradrenaline release from sympathetic nerve terminals [71].

Endocannabinoid-Mediated Regulation of the Hypothalamus-Pituitary-Adrenal Axis

The HPA axis constitutes the principal circuit responding to exercises and other stress-inducing situations [70,72]. Upon encountering aerobic exercise, neurons within the hypothalamic paraventricular nucleus (PVN) release substantial quantities of corticotropin-releasing hormone (CRH) into the portal vessels of the median eminence. Within the pituitary gland, CRH instigates adrenocorticotropic hormone (ACTH) secretion, thereby setting the synthesis and subsequent release of glucocorticoids, primarily cortisol, from the inner adrenal cortex into the bloodstream.

Cortisol, once secreted, orchestrates the rapid mobilization of stored energy reserves, orchestrating a countermeasure against the stresses induced by exercise. Notably, cortisol prompts the swift generation of eCBs within hypothalamic neuroendocrine cells via the activation of CB_1_R. This expedited eCB synthesis is evoked fleetingly through robust post-synaptic depolarization and the entry of Ca^2+^ ions via voltage-gated Ca^2+^ channels [73,74], triggered by synaptic activity [75-77] and G protein-coupled receptor stimulation linked to phospholipase C (PLC) signaling [73,78]. 

**Figure 4:**
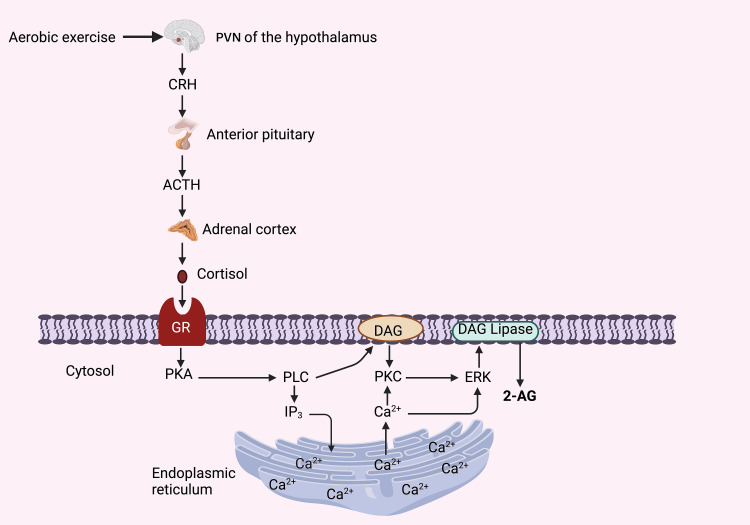
Illustration of the signaling cascade governing cortisol-induced endocannabinoid (2-AG) synthesis. Notes: This intricate process can transpire during various stress-inducing scenarios, including aerobic exercise. However, the knowledge concerning the membrane-associated glucocorticoid receptor (GR) remains rather enigmatic. This entity could manifest as either the nuclear glucocorticoid receptor at the membrane's interface or an undiscovered G protein-coupled receptor [81]. PVN: Paraventricular Nucleus, CRH: Corticotropin-Releasing Hormone; ACTH: Adrenocorticotropic Hormone, DAG: Diacylglycerol; 2-AG: 2-Acylglycerol, PKA: Protein Kinase A, PLC: Phospholipase C; IP3: Inositol Triphosphate, PKC: Protein Kinase C, ERK: Extracellular Signal-Regulated kinase. This figure has been drawn with the premium version of BioRender (https://www.biorender.com/ Accessed date January 25^th^, 2024)with License Agreement No.: IO26DRZFV4. Image Credit: Subir Gupta

It has also been documented that eCB synthesis necessitates the involvement of protein kinases. Specifically, cAMP-dependent protein kinase (PKA) and protein kinase C (PKC) have both emerged as contributors to eCB production [79, 80]. However, the precise role of kinase activity in eCB release remains a subject of ongoing elucidation. A comprehensive model detailing cortisol-induced eCB synthesis has been presented in Figure 4.

A Threshold of Exercise Intensity Prerequisite for Cortisol Secretion

The initiation of cortisol secretion from the adrenal glands appears to be contingent upon surpassing a threshold in exercise intensity. Numerous studies have concurred that the minimal exercise intensity requisite to trigger a cortisol response is about 60% of VO_2_ max or entails engaging in moderate activity during submaximal exercise [82-84]. Once this crucial threshold is surpassed, a direct and positive linear correlation emerges between cortisol output and exercise intensity, with a notable and significant escalation in cortisol secretion observed at higher submaximal work rates [83-85].

Endocannabinoid-Mediated Negative Feedback on the HPA Axis

Excess cortisol secretion triggers the negative fast feedback mechanism upon the HPA axis. This prompts the biosynthesis of eCBs, activating CB_1_R in glutamate terminals. Consequently, this reduces or suppresses glutamate release and inhibits further retrograde production of CRH. Additionally, negative fast feedback in the amygdala is facilitated through eCBs signaling in the BLA-GABAergic outflow [86]. Cortisol secretion operates through both direct and indirect pathways. The direct feedback transpires at the hypothalamic and pituitary levels [87-89]. Indirect inhibition by cortisol within the HPA axis is orchestrated by upstream limbic structures such as the hippocampus, paraventricular thalamus, amygdala, prefrontal cortex (PFC), and bed nucleus of the stria terminalis (BNST), all of which project onto the CRH secreting neurons of the hypothalamic paraventricular nucleus (PVN) [72,90,91]. Outputs stemming from the PFC and hippocampus/subiculum encompass excitatory projections that traverse to the PVN via principal neurons, wherein their direction is reversed through inhibitory relays situated within the BNST and peri-PVN hypothalamic regions [72,91]. In detrimental feedback modulation of the HPA axis, aerobic exercise (and other physiological stressors) primarily leverages the fast direct pathway. In contrast, psychological stress predominantly influences the axis through the slower, higher limbic structures.

The distribution of CB_1_R is particularly prominent in limbic structures and subcortical regions, including the BNST mentioned above and PVN. Furthermore, CB_1_Rs are situated within these structures at the presynaptic sites of GABAergic and glutamatergic synapses, activating inhibitory effects on neurotransmitter release [92]. Although CB_1_Rs are expressed to a lesser extent in glutamatergic neurons compared to GABAergic neurons, the decrease in glutamate release from glutamatergic synapses due to the activation of CB_1_Rs plays a critical role in the glucocorticoid-mediated feedback regulation of the HPA axis [93].

Notably, CB_1_Rs are also found in the intermediate and anterior lobes of the pituitary gland, as well as in the outer region of the median eminence [94-96]. Additionally, eCBs 2-AG and AEA have been identified within the pituitary gland [95]. Given that CB_1_Rs are also present in the adrenal gland [97], a cumulative body of evidence underscores the pivotal regulatory role of CB_1_Rs, as opposed to CB_2_Rs, in governing the HPA axis.

**Figure 5:**
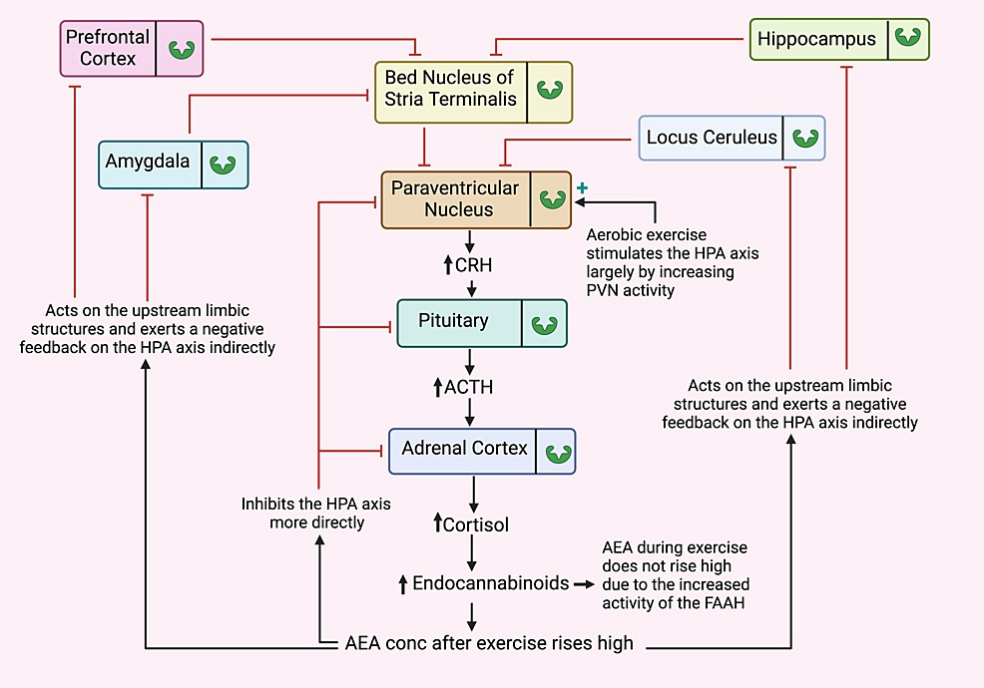
Schematic depiction of aerobic exercise's impact on the hypothalamus-pituitary-adrenal (HPA) axis. Notes: Aerobic exercise triggers HPA axis stimulation, primarily emanating from the hypothalamic paraventricular nucleus (PVN), culminating in the heightened secretion of cortisol and endocannabinoids. During the recovery phase, a notable elevation in endocannabinoid levels, particularly anandamide (AEA), engenders an indirect suppression of the HPA axis through the engagement of upstream limbic structures. Furthermore, endocannabinoids execute a more direct inhibitory influence on the HPA axis by acting upon the hypothalamus, pituitary, and adrenal cortex. While engaged in exercise, the elevation of AEA levels is hampered by the catabolic role of fatty acid amide hydrolase (FAAH) on AEA). Notably, all the limbic and sub-limbic structures illustrated in this diagram house Cannabinoid Receptor Type 1 (CB_1_R), denoted by underscoring their integral involvement in this intricate cascade. CRH: corticotropin-releasing hormone, ACTH:  adrenocorticotropic hormone. This figure has been drawn with the premium version of BioRender (https://www.biorender.com/ Accessed date January 25th, 2024)with License Agreement No.: EM26DRZM3F. Image Credit: Ambadasu Bharatha

* *Figure 5 illustrates aerobic exercise's impact on the hypothalamus-pituitary-adrenal (HPA) axis.

Tonic Inhibition of the HPA Axis Stress Response via CB1R Signaling

The presence of a stressor correlates with sustained suppression of AEA levels. This stems from the rapid surge of CRH signaling in limbic structures upon acute stress exposure, which augments the enzymatic activity of FAAH (Figure 5). Consequently, there is a swift attenuation of AEA's inhibitory influence on the HPA axis [9,50].

Physical exercise induces a surge in cortisol, which spurs the generation of 2-AG in the hypothalamus and other stress-associated brain regions, thus elevating CB_1_R signaling. This, in turn, fosters negative feedback inhibition of the HPA axis, thereby facilitating the cessation of the stress response [9]. 

Aerobic training or sustained exercise elicits a progressive surge in 2-AG levels within forebrain stress centers. This heightened 2-AG concentration subsequently amplifies CB_1_R signaling, culminating in the habituation of the HPA axis response [9,98]. The elevation in 2-AG production may be attributed to the downregulation of MAGL expression.

Possible brain neural reward circuit associated with aerobic exercise

The ECS plays a significant role in signaling rewarding events. This is primarily attributed to the presence of CB_1_R in brain regions implicated in reward processes and the alteration of brain levels of AEA and 2-AG due to the activation of reward mechanisms [99-101]. Endocannabinoids directly engage with the reward system, endowing them with addictive properties. The reinforcing effects of eCBs stem from their ability to heighten tonic dopamine levels through a CB_1_R-dependent mechanism within the ventral tegmental area (VTA) [102]. 

**Figure 6:**
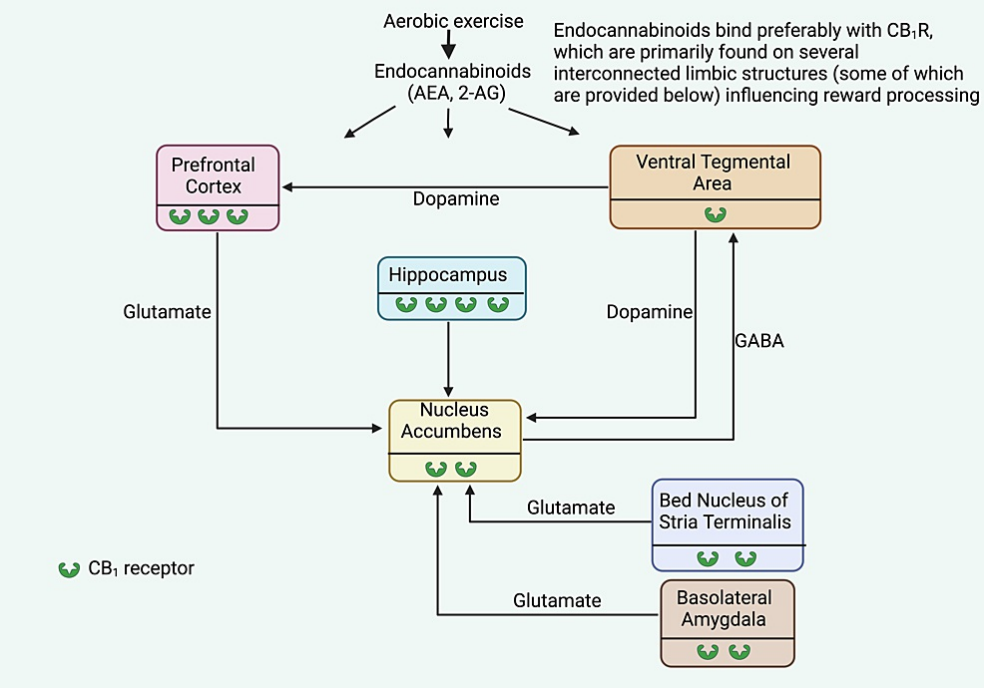
Potential reward pathways in the brain Notes: The presence of CB_1_R, denoted by, is evident across diverse limbic regions, as elucidated in this presentation. The quantity serves the degree of CB_1_R abundance – a single denotes an exceedingly limited concentration, whereas a cluster of four signifies a markedly elevated concentration [105]. PFC: Prefrontal cortex, VTA: Ventral Tegmentum; HIPP: Hippocampus, NAc: Nucleus Accumbens; BNST: Bed Nucleus of Stria Terminalis, BLA: Basolateral Amygdala, GABA: Gamma-aminobutyric acid. This figure has been drawn with the premium version of BioRender (https://www.biorender.com/ Accessed date January 25th, 2024) with License Agreement No.: HV26DRZOKF. Image Credit: Ambadasu Bharatha

The ECS-mediated reward processing predominantly operates through a network of interconnected structures, encompassing the nucleus accumbens (NAc, also referred to as ventral striatum), VTA, prefrontal cortex (PFC), amygdala, and bed nucleus of the stria terminalis (BNST). Beyond dopamine's well-established role, reward processing is substantially influenced by other systems, such as the cholinergic, opioid peptide, glutamatergic, and GABAergic systems. CB_1_Rs are present in all interconnected structures implicated in reward processing [42,94,101,103], exerting pervasive modulatory effects on excitatory and inhibitory signaling, thereby shaping reward processing dynamics [100,104]. Particularly notable is the ECS's profound involvement in refining the activity of the VTA-NAc dopamine projection, thereby influencing approach and avoidance behaviors governing reward acquisition (Figure 6) [105].

The mesocorticolimbic dopamine pathways, emanating from the midbrain VTA, play a pivotal role in mediating reward responses. In particular, the VTA's dopamine projection to the NAc prominently contributes to positive reinforcement, resulting in reward acquisition. Aerobic exercise and other natural rewards (such as food, sex, and substances of abuse) elicit an elevation in NAc dopamine levels, contributing to the subjective experience of reward and positive reinforcement [106]. VTA dopamine neurons also innervate additional limbic system components, encompassing the amygdala, hippocampus, orbitofrontal cortex, and specific prefrontal cortex regions. These intricate circuits involve both excitatory (mainly glutamatergic) and inhibitory (primarily GABAergic) projections [107]. 

Dopaminergic neurons exhibit two distinct modes of activity: tonic and phasic firing [108]. Tonic activity entails pacemaker-like spontaneous single spikes, while phasic activity is characterized by rapid, transient surges in dopamine levels resulting from high-frequency bursts [108]. Phasic dopamine neuron activity is essential for establishing enduring memories that link predictive cues with rewards. In contrast, the tonic activity of these neurons governs the motivation to respond to such cues [109]. An appropriate emotional response to stress is imperative for survival and necessitates precise regulation of diverse neuronal circuits. Thus, meticulous control of these circuits is vital to avert behavioral imbalances.

Over the past two decades, extensive research has highlighted the ECS's critical role in regulating stress-coping mechanisms [110]. The ECS is known to oversee neurotransmitter release from various neuron populations (e.g., GABA, glutamate, catecholamines, and monoamines), suggesting a fundamental mechanism for fine-tuning neuronal activity and thereby regulating emotional and stress responses [110]. 

Discussion

Substantial evidence underscores the noteworthy involvement of eCBs in eliciting a favorable shift in mood after acute episodes of exercise. However, the intricate mechanisms underpinning the eCB-mediated mood enhancement following aerobic exercise remain inadequately explored. Additionally, the upliftment in mood that comes from exercise is influenced not just by the release of eCBs but can also happen through different psychological or environmental factors, such as the context and surroundings in which the exercise occurs [2].

Aerobic exercise precipitates a substantial surge in eCBs originating from diverse sources, which can permeate the blood-brain barrier, exerting their influence at multiple cerebral sites housing eCBRs. Notably, moderate to high-intensity aerobic exercise induces a substantial increase in cortisol secretion, consequently fostering heightened eCB production. This surge of eCBs orchestrates a negative feedback control mechanism over the HPA axis, thus effectively regulating stress levels. Lowering eCB levels through physical training will likely result in physiological adaptations that decrease the essential pro-inflammatory state. Consequently, exercise training might influence the plasma levels of eCBs and their analogs, such as how it affects other inflammatory molecules [54].

The elevation of eCBs likely mitigates sympathetic stimulation, although the precise mechanisms behind this phenomenon remain enigmatic, ultimately contributing to further stress alleviation. Moreover, the efficacy of the ECS extends to stimulating the brain's reward circuitry, primarily through the activation of CB_1_R strategically positioned across various cerebral locales, each employing distinct neurotransmitters.

Although an array of receptor-specificities distinguishes AEA and 2-AG, both eCBs are synthesized responsively, predominantly triggered by elevated intracellular calcium concentrations [48]. However, synthesis, transport, and deactivation processes for AEA and 2-AG vary across their specific target tissues. A persistent conundrum in eCB research pertains to how lipophilic AEA traverses its synthesis site, navigates the aqueous milieu, and arrives at diverse intracellular membrane areas where its metabolic and signaling activities occur [111,112]. Intricacies in studying intracellular AEA transport and distribution stem from the lack of tailored probes and techniques to track and visualize this bioactive lipid within cellular confines [111,112].

While many investigations have focused on walking or jogging regimens, the efficacy of resistance exercise has also been explored. In the context of depressed elderly individuals, resistance training demonstrated greater effectiveness compared to a control condition [5]. In a comparison between random assignments to running or weightlifting, Doyne et al. [113] noted that both activities led to diminished depressive symptoms, with no statistically significant discrepancies at the culmination of the active treatment phase or during a subsequent one-year follow-up. Similarly, Martinsen et al. [4] discovered no discernible disparities between aerobic activities (such as jogging or brisk walking) and non-aerobic forms of exercise (including strength training, coordination, and flexibility training).

The brain's reward system stands as a pivotal survival mechanism. The hedonic effects of aerobic exercise hold pivotal motivational implications, enhancing the likelihood of future engagement in these vital activities through positive reinforcement. The mood-enhancing role of eCBs during aerobic exercise holds evolutionary significance for species survival. Yet, not all intensities of aerobic exercise are equally effective in generating eCBs and their subsequent psychological well-being effects. Moderate-intensity exercise outperforms both excessively high and excessively low intensities in this regard.

While the precise neurophysiological mechanism driving exercise-induced mood alterations and reductions in anxiety and depression remains elusive, a distinct neural circuit within the limbic cortex appears paramount. The brain's reward circuit forms an intricate network comprising diverse neural centers such as the hippocampus, amygdala, VTA, nucleus NAc, PFC, and BNST. All these reward circuit components harbor CBRs and release an array of neurotransmitters, with dopamine, glutamate, and GABA emerging as the most prevalent. Notably, CB_1_R exhibits a broader distribution than CB_2_R, and eCBs influence CB_1_R on presynaptic neurons, yielding comprehensive modulatory effects on excitatory and inhibitory signaling, thereby shaping reward processing dynamics [100,104]. Endocannabinoids feature prominently in fine-tuning the VTA-NAc dopaminergic projection's activity, thereby shaping behaviors related to seeking rewards and avoiding adverse outcomes.

Following an extended run, AEA and 2-AG trigger a state of euphoria, colloquially referred to as the "runner's high" [1], linked to the central effects of circulating eCBs, which enhance the hedonic signal within the brain's reward system, fostering a proclivity for regular aerobic exercise. Moreover, elevated eCB levels during exercise amplify brain-derived neurotrophic factor (BDNF), mediating cognitive benefits such as neurogenesis, synaptic plasticity, and antidepressant effects [1,51]. These central effects, combined with peripheral outcomes like improved glucose uptake, enhanced insulin action, and mitochondrial biogenesis, underscore the clinical and scientific significance of comprehending eCBs responses to exercise, especially in investigating the requisite exercise intensity for eliciting such effects [1,51]. However, controversies persist regarding the necessary exercise intensity to achieve this effect [56,114,115].

Aerobic exercise demands an intensity of at least 60% of VO_2max_ to evoke a consistent cortisol response [116]. The HPA axis is a pivotal target of eCB, selectively restraining the axis and maintaining controlled cortisol levels, particularly evident in both aerobically trained individuals and acute exercise scenarios. Psychological stress exerts a gradual negative feedback loop through limbic structures on the HPA axis. However, this mechanism's efficiency remains limited, and frequent uncontrolled psychological stress may fail to mitigate cortisol elevation, potentially resulting in hypertension and disorders related to excessive cortisol levels. During aerobic exercise, the upsurge of eCBs directly inhibits the HPA axis through CB_1_Rs present in the hypothalamic PVN, pituitary gland, and even the adrenal cortex. Indications suggest that AEA and 2-AG play pivotal roles in this feedback process [95].

Exercise positively impacts cognitive performance [117], influencing the brain's reward system [118] and evoking a sense of contentment. By modulating multiple signaling mediators, exercise effectively impacts mood [117] and nociception [119], influencing immune system functions [120] alongside comprehensive whole-body energy metabolism [121]. Endocannabinoids mediate both central and peripheral effects of exercise that contribute to psychological well-being [122].

Future research

The following avenues of research demand exploration to architect tailored exercise protocols suited to diverse demographic groups, encompassing age, gender, familial, and societal contexts: i) Delving into the effects of intermittent aerobic exercise on the eCBs and its influence on mood elevation holds profound significance. The challenges many adults face in sustaining medium-intensity aerobic regimens necessitate a deeper understanding of the impact of rest intervals between exercise bouts, potentially extending exercise duration. ii) Scrutinizing the role of sprinting, fast runs, or high-intensity, short-duration exercises holds potential for healthy adults and individuals afflicted by mood disorders. This holds particular relevance for younger populations and those endowed with exceptional physical fitness. iii) Exploring the effects of recreational team sports is an uncharted terrain. Addressing the formidable motivation challenge, particularly among adults, is critical. Team sports like football, hockey, or basketball may serve as compelling outlets, yet their intermittent nature and potential impact on mood elevation warrant rigorous investigation. iv) Comparing the efficacy of home-bound exercise, such as treadmill running or stationary cycling, with outdoor activities emerges as an intriguing subject. Given individuals' time constraints, particularly women and busy professionals, deciphering whether indoor exercises yield commensurate benefits is essential. v) Investigating whether an individual's preferred exercise mode, cycling or swimming, engenders a more potent reward response than non-preferred alternatives holds pivotal insights. Acknowledging the diverse preferences that individuals harbor understanding the interplay between exercise modes and rewarding outcomes has implications for adherence and sustainability. An unwavering commitment to rigorous inquiry and systematic exploration is warranted to chart the uncharted terrain. The intricate intersections of aerobic exercise, the eCBs, and psychological well-being beckon for illumination, promising innovative interventions that could transform the lives of those grappling with multifaceted health challenges.

## Conclusions

The connection between aerobic exercise and eCBs regarding stress involves the exercise-induced elevation of eCB levels, potentially contributing to the stress-relieving effects of physical activity. Numerous aspects of the interplay between aerobic exercise, the ECS, and psychological well-being remain obscured. Nevertheless, physiologically significant insights gleaned from research warrant attention and elucidation. a) Following a session of aerobic exercise, the blood concentration of AEA demonstrates an upsurge, with the magnitude of this increase contingent upon the exercise's intensity. Notably, moderate-intensity aerobic exercise incites a substantial elevation in AEA levels. Conversely, the post-aerobic exercise plasma levels of 2-AG remain subject to debate. b) The sensation of euphoria often associated with running finds partial explanation in heightened circulating AEA levels. Generally, a single bout of aerobic exercise is correlated with sustained reductions in state anxiety and physiological arousal, persisting for 2 to 4 hours. c) Elevated eCB levels consequent to exercise also emerge as a pivotal contributor to the augmentation of BDNF, thereby facilitating cognitive enhancements, neurogenesis, synaptic plasticity, and even antidepressant effects. d) The ECS's pivotal role in mediating the effects of aerobic exercise primarily revolves around CB_1_R activation. While CB_2_R is limited in mood elevation, CB_1_R is paramount. e) Notably, substantial psychological enhancements have been observed in individuals experiencing moderate depression after engaging in consistent exercise. However, alterations in individuals within the typical spectrum of depression and anxiety are either modest or absent from observation. f) The presence of eCBRs is noted within the PVN of the hypothalamus, along with the anterior pituitary gland. Acute aerobic exercise engenders negative feedback within the HPA axis by elevating both AEA and 2-AG levels. g) The ECS assumes a critical role in signaling rewarding effects. Endocannabinoids orchestrate these effects via interaction with CB_1_R distributed across diverse regions of the limbic cortex. Alongside dopaminergic neurons, the intricate landscape of reward processing is also significantly contingent on cholinergic, GABAergic, and glutamatergic neurons. The limbic centers implicated include the VTA, NAc, BNST, and PFC. Notably, eCBs play a crucial role in fine-tuning the function of the VTA-NAc dopaminergic pathway, thus influencing behaviors related to seeking rewards and avoiding adverse outcomes.
